# Rhythmic Jaw Movements in Amyotrophic Lateral Sclerosis: Is It Clonus or Tremor?

**DOI:** 10.5334/tohm.845

**Published:** 2024-02-29

**Authors:** Rohini Kumar, Jamie Blackband, Aparna Wagle Shukla

**Affiliations:** 1 Department of Neurology, Norman Fixel Institute for Neurological Diseases, University of Florida, Gainesville, Florida, United States of America

**Keywords:** clonus, tremor, amyotrophic lateral sclerosis (ALS), stretch reflex, jaw clonus

## Abstract

**Background:**

Jaw clonus refers to involuntary, rhythmic jaw contractions induced by a hyperactive trigeminal nerve stretch reflex; however, the movements, when triggered without a stretch, can be confused with a tremor.

**Phenomenology Shown:**

This video demonstrates a patient with amyotrophic lateral sclerosis presenting with rapid rhythmic jaw movements seen at rest, alongside a power spectrum analysis revealing a narrow high-frequency peak of 10 Hz.

**Educational Value:**

Rhythmic jaw movements are seen in many disorders such as Parkinson’s disease, essential tremor, tardive syndromes, and cranial myorhythmias; however, a high-frequency movement, regardless of clonus or tremor, can indicate amyotrophic lateral sclerosis when accompanied by typical upper and lower motor neuron signs.

**Highlights:**

The presented video abstract shows a patient with amyotrophic lateral sclerosis with rhythmic jaw movements seen at rest. A power spectrum analysis of the rhythmic movements revealed a 10 Hz peak, a frequency higher than those seen in patients with Parkinson’s disease, essential tremor, myorhythmia, and tardive syndromes.

Jaw clonus refers to involuntary, rhythmic contractions of the jaw muscles induced by a hyperactive trigeminal stretch reflex. Jaw clonus is a known clinical feature of amyotrophic lateral sclerosis (ALS), albeit rare, and the rhythmic movements are generally triggered when the jaw is activated. Rhythmic jaw movements are observed in many neurological disorders, such as Parkinson’s disease, essential tremor, tardive syndromes, and cranial myorhythmias [1,2]. Rhythmic jaw movements could be observed in patients with enhanced physiological anxiety.

A 55-year-old white man presented with rapid worsening of speech and swallowing functions. He described his speech as slow and slurred with a strained vocal quality. He complained of coughing and choking episodes when eating hard and solid foods. He felt his tongue had become thick. He began to experience spasms in the jaw muscles accompanied by jaw shaking that was intermittent initially, observed during chewing, speaking, and swallowing, but at the time of clinical presentation, it was seen at rest as constant movement. Within months, there was arm weakness and frequent muscle twitching. A physical exam revealed a fast-rhythmic movement of the jaw [Video 1, left panel], mild jaw weakness, brisk jaw jerk, and fasciculations seen in the tongue and arm muscles. Based on clinical presentation and electrodiagnostic testing, he was diagnosed with bulbar onset amyotrophic lateral sclerosis. A power spectrum Fourier analysis of the accelerometer recordings from the masseter muscles revealed a 10 Hz peak [Video 1, right panel] for the rhythmic jaw movements consistent with the previous literature that found the oscillation frequency to range from 7.5 to 15 Hz [3]. The patient could not be followed longitudinally for a trial of anti-spasticity agents as he died within 3 months of first visit and could not return for a follow-up visit.

**Video 1:** **Rhythmic Jaw Movements in ALS.** The video demonstrates rhythmic jaw movements observed with the mouth closed in the left panel. Physiological recordings were obtained by placing a sensor over the masseter muscle, as shown in the right panel. A power spectrum analysis revealed a peak frequency of 10 Hz for the rhythmic movements.

Clonus is related to self-excitatory hyperactive stretch reflexes seen in the presence of upper motor neuron dysfunction. Each time the muscle relaxes from the previous reflex contraction, the applied stretching force renews the reflex, setting up a rhythmic series of muscle contractions that continue as long as the tension is applied. In the case of jaw clonus, jaw opening or elicitation of jaw jerk would be expected to activate the stretch reflex. In our patient, the rhythmic jaw movements were constantly seen and did not require elicitation of a jaw jerk. We speculate that ALS related weakness of the masseters led to a slight, constant opening of jaw and alteration of muscle tension. As a consequence, stretching of muscle spindles in conjunction with a hyperactive jaw jerk led to self-perpetuating cycle of rhythmic movements. It is possible that in early ALS, elicitation of jaw jerk is a prerequisite to observe a jaw clonus. A lack of longitudinal tracking of the clinical phenomenon limits the current case description. The movement frequency for clonus movements varies inversely with the length of the reflex path, with higher frequencies expected in the jaw, given the shorter path compared to the wrist and the ankle. Some have proposed that similar to tremor, central oscillators may be activated even in clonus, leading to rhythmic stimulation of the lower motor neurons [1]. Although the pathophysiology appears to overlap considerably, the rhythmic jaw movement of ALS is more likely a clonus and not a tremor. Electrophysiological assessment of the masseter inhibitory reflex could be pursued to understand the excitability of the brainstem and the cortex. In this assessment, stimulation response to mandibular nerves is recorded with EMG of the jaw muscles. Nerve stimulation is known to elicit two periods of muscle silence: first or early component reflecting the shorter oligosynaptic spinal pathway and the second reflecting the longer or long-loop transcortical reflex [4]. Latencies and durations of the silent periods are able to reveal the excitability of the brainstem and the sensorimotor system. We speculate that in the setting of ALS, the early component of the silent period would likely be shortened indicating an increased excitability of the trigeminal reflex system.

From a differential diagnosis standpoint, the rhythmic jaw movements can be seen in enhanced physiological anxiety however these patients likely have a fast and fine tremor in many other body parts. In Parkinson’s disease, jaw tremor seen at rest has a low frequency (4–6 Hz) whereas in essential tremor these movements are triggered by mouth opening or closing when talking or chewing, and importantly, there is a co-occurring arm tremor. Myorhythmia affecting the jaw in Whipple’s disease and NMDA encephalitis is generally slow (1–4 Hz), and tardive jaw tremor commonly co-occurs with lip and chin movements. In a nutshell, there is little research on rhythmic movements affecting the jaw. A head-to-head comparison between rhythmic jaw movements in ALS patients and those in other neurological conditions is warranted to provide valuable insights.
